# Supplementary material for: Bayesian modeling of HFC production pipeline suggests growth in unreported CFC by-product and feedstock production
Source: Nat Commun. 2024 Dec 30;15:10883. doi: 10.1038/s41467-024-55250-6 (PMC11685568; doi:10.1038/s41467-024-55250-6)
Supplement: Supplementary file 1 — Supplementary Information [file 41467_2024_55250_MOESM1_ESM.pdf]

Supplementary information for *Bayesian modeling of HFC production pipeline suggests growth in unreported CFC by-product and feedstock production*

Stephen Bourguet[1*] and Megan Lickley[1,2]

1 Earth Commons, Georgetown University, Washington, DC 20057, USA

2 Science, Technology, and International Affairs Program, Georgetown University, Washington, DC 20057, USA

***Corresponding author: stephen.bourguet@georgetown.edu**

Supplementary Note

HFC production

To support our assumptions regarding CFC and HCFC emissions during HFC production, here we review the possible points of CFC emissions during the production of HFC-134a and HFC-125. These HFCs comprised approximately two thirds of HFC emissions by mass from 2015–2019 (not including HFC-23, which has minimal intentional use and is controlled separately under the Montreal Protocol) [1]. We also highlight the production of HCFC-133a, which we use as an additional constraint on the production of HFC-134a and HFC-125. The production pipeline of these two HFCs is summarized in Figure 2 of the main text and informs our assumptions in our modeling framework (see Methods).

HFC-134a

HFC-134a is the most widely used HFC [1]. Its production can be achieved by either of two pathways [2] that are distinguished by their unsaturated feedstocks, trichloroethylene (TCE) and perchloroethylene (PCE), as is shown in Figure 2 of the main text. For pathway 1, TCE has one hydrogen atom (chemical formula: C_2HCl_3), so the intermediate products in the reactions that yield HFC-134a are HCFCs [2, 3]. In particular, HCFC-133a is an intermediate of this process with no known direct end-use [4], making it a valuable constraint in our modeling framework. For pathway 2, PCE has a chlorine atom in place of the hydrogen atom (chemical formula: C_2Cl_4), so the intermediates during fluorination may be CFCs and HCFCs [2, 5, 6]. Therefore, CFC-113, CFC-113a, CFC-114, or CFC-114a is only produced and emitted during HFC-134a production if the PCE route is used. Unless otherwise noted, we refer to the sum of isomers by the major isomer throughout this text (e.g., CFC-113+CFC-113a = CFC-113).

Previous reports suggest that the TCE/HCFC-133a pathway accounts for the majority of HFC-134a production [7, 8, 9], but the exact division of these two pathways, as well as the possible geographic distribution of which pathway is used, has not been reported. In addition, previous work has shown that impurities in the emission of HFC-134a at the point of consumption (i.e. leakage from air conditioning units) cannot explain the recent emissions of CFC-113 or CFC-114 [10], leaving the production process as the primary source of HFC-134a-related CFC emissions.

A bottom-up estimate of the division of production between the two pathways could be calculated with proper accounting of the HCFC-133a, CFC-113, or CFC-114 molecules consumed in HFC-134a production; however, feedstock reporting requirements only apply when these compounds are produced and consumed in separate processes, allowing for the potential for production to lawfully go unreported. Additionally, countries may simply not be reporting their production of these compounds despite their agreement to do so, as was the case with recent CFC-11 and CFC-12 emissions [11, 12]. Between 2008 and 2020, non-Article 5 (high income) countries reported feedstock production of CFC-113 and 114 on the order of 3000 Gg, but Article 5 (low to middle income) countries did not report CFC-113 feedstock production during this time [13], despite an estimated quadrupling of their HFC-134a production (Figure 1C of the main text, [1]). It is unlikely that A 5 countries did not use CFC-113 in the production of HFC-134a during this time (if they exclusively produced HFC-134a using the TCE pathway) – within this time period, there was a reported enhancement of CFC-113a and CFC-114a measured in air samples collected downwind of a region where HFC-134a is produced in China [14], suggesting that some amount of HFC-134a produced in that region used CFC-113a and CFC-114a as a feedstock. There also was not a rise in global HCFC-133a emissions during this time [15], which would have been expected if the new HFC-134a production was manufactured using the TCE pathway.

On the other hand, a top-down inference of the production of HFC-134a by each pathway could be calculated using observed mixing ratios of HCFC-133a, CFC-113, and CFC-114 if there was complete knowledge of the chemical conversion rates and emission rates of the relevant production processes, along with well-constrained bank emission estimates. However, there are large uncertainties in conversion and emissions rates and published patents suggest that these rates may have varied temporally. Specifically, the production pathway that uses HCFC-133a as an intermediate has a yield rate that could vary by a factor of three: If the HCFC-133a to HFC-134a reaction is run once, then the yield rate is limited to about 30%, but if the output from this reaction is run through a second reactor, then the yield rate increases to over 90% [2, 3]. Thus, for a given HCFC-133a emissions profile, the amount of HFC-134a produced from HCFC-133a could vary by at least a factor of three, and uncertainty in the emission rate would increase this range of possibilities further. The link between CFC-113 and

CFC-114 emissions and their production is also uncertain. MCTOC 2022 reported the "most likely" range of the total emission rate from the production, transport, and consumption of feedstocks to be 1.5–6.2% [17], and the conversion rates for each step in the process from CFC-113 to HFC-134a depend on the catalyst employed as well as whether CFC-113 is first fluorinated or isomerized [2], or if both steps are carried out in one reaction. This published range of possible emission and conversion rates thus makes top-down emissions attribution infeasible.

HFC-125

During the production of HFC-125, an undesirable side reaction can occur that yields CFCs as by-products rather than the intended HCFC intermediates and HFC-125 [16, 17]. Depending on the extent of fluorination, CFC-113, CFC-114, and/or CFC-115 may be produced by this side reaction, though CFC-115 has been identified by relevant patents as the primary by-product [16, 18]. CFC-115 and HFC-125 are both the most fluorinated species in their reaction chains, so less fluorinated CFCs should be converted to CFC-115 during the fluorination process. Additionally, HCFC-133a is an undesirable by-product of the main reaction chain [16].

In a 2015 patent for HFC-125 production, it was reported that 1.1%, 1.0%, 2.1%, and 1.1% of the PCE feedstock could be converted to CFC-113, CFC-114, CFC-115, and HCFC-133a on a molar basis, respectively [16]. The CFC-115 by-product emission rate has also been estimated based on plant data as 0.1-1 wt%, while other controlled substances were assumed to be emitted at rates one or more orders of magnitude smaller [18]. Technology exists to separate the undesired CFCs from HFC-125 [19], although the extent to which this technology is employed has not been well-documented. Moreover, for the CFCs that are separated from high purity HFC-125, the extent to which they are destroyed or released into the atmosphere is not reported. Thus, both the mass of these by-products generated and their emission rates from this process are uncertain, so only the total emission rate relative to total HFC-125 production can be estimated.

As was the case with HFC-134a, there is an additional production pathway for HFC-125 that does not produce CFCs, which involves the hydrofluorination of tetrafluoroethylene (TFE) with HF [20]. TFE and HF are devoid of chlorine, so CFCs will not be produced during this process. There is limited information on which production process is used, but a 2017 report from the Chinese Chemical Investment Network Stated that only 4 out of the 12 factories in China that produce HFC-125 used the PCE pathway in 2011 [10]. Outside of China, the allocation between these pathways has not been made publicly available, although it was reported in 2023 that "most" HFC-125 was produced via PCE [18]. There is not an observational proxy in the atmosphere for production via the TFE pathway, so it is only possible to compute emissions relative to total HFC-125 production and treat this rate as a lower bound.

Supplementary Methods

Lifetime sensitivity test

CFC lifetimes are key parameters in emission inversions [21] and in our simulation model (see Methods). We test the sensitivity of our results to a range of lifetimes informed by the Stratospheric Processes and their Role in Climate (SPARC) report on the Lifetimes of Stratospheric Ozone-Depleting Substances, Their Replacements, and Related Species [22]. This report provides "most likely" lifetime ranges for CFC-11, CFC-12, and CFC-113, but not for CFC-114 and CFC-115. We assume that the "most likely" range of CFC-114 and CFC-115 lifetimes scales with that of CFC-12 and CFC-113, as these compounds have overlapping stratospheric loss regions [22]. The suggested lifetimes of CFC-113, CFC-114, and CFC-115 are 93, 191, and 540 y, respectively, and the "most likely" ranges of CFC-12 and CFC-113 are about 88–118% of their median values. Thus, we assume that the lifetimes of CFC-114 and CFC-115 also vary by 88–118% of their median values, and we test the sensitivity of our results with CFC-113, CFC-114, and CFC-115 lifetimes of 82–109, 168–225, and 475–635 y, respectively.

Supplementary Figures

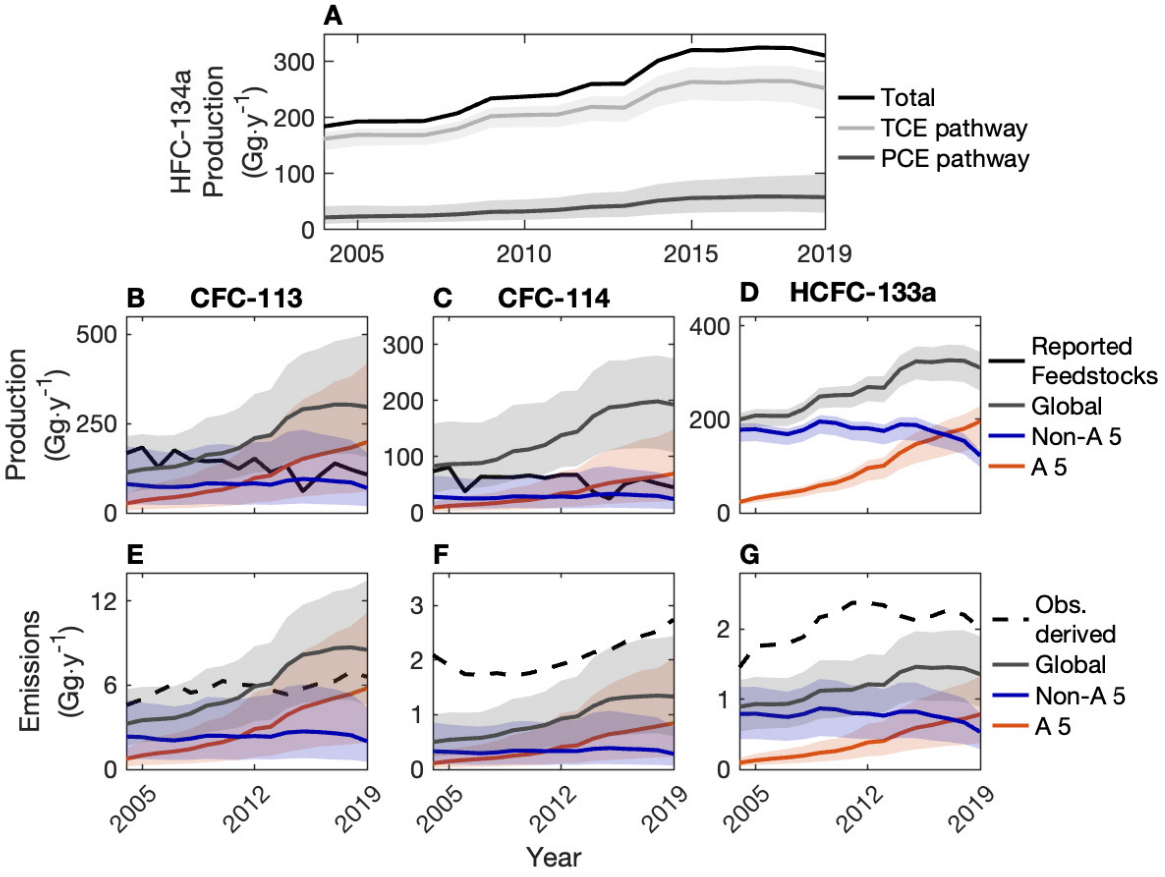

Supplementary Figure 1: Same as Fig. 4 in the main text, but with the CFC-113 to CFC-114 chemical conversion rate reduced to 40% [2]. (A) Estimated global HFC-134a production (black; data from [1]) and the BPE estimated mass of HFC-134a produced using TCE (light gray) and PCE (dark gray) as feedstocks. BPE posterior distributions of (B–D) production and (E–G) emissions of CFC-113 (left), CFC-114 (middle), and HCFC-133a (right). The lines and shaded regions are the median and 1-σ CI, respectively, and in B–G, the gray, blue, and orange coloring denotes global, non-A 5, and A 5 countries, respectively. For reference, the mass of feedstock production reported to the Ozone Secretariat [23] is included in B and C, and the 5-y running means of observationally-derived emissions are included in E–G. Note the increase in estimated CFC-113 production in (B) relative to Fig. 4 in the main text. (Also note the increased range on the y-axis in (B), (D), and (E) relative to Fig. 4.)

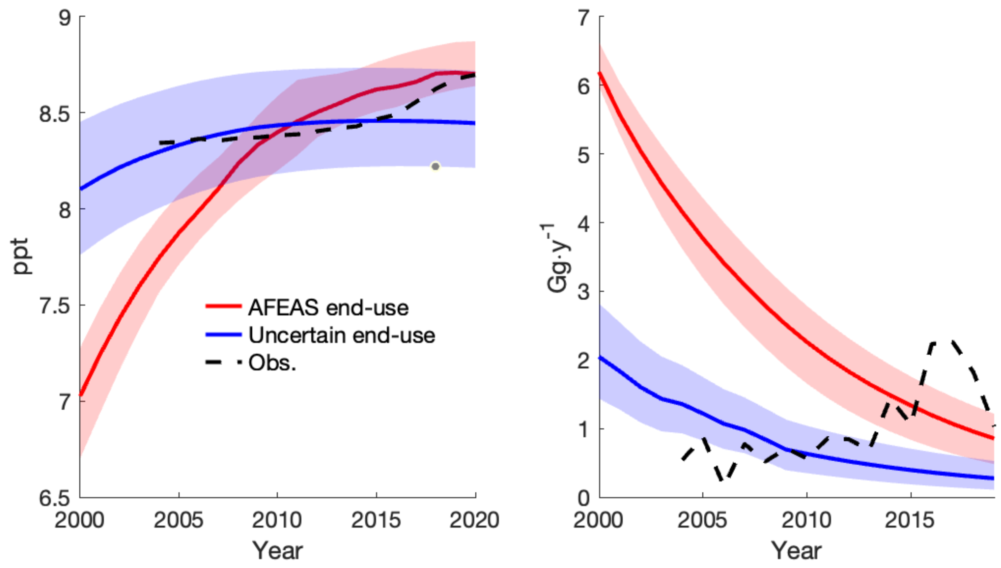

Supplementary Figure 2: BPE posterior CFC-115 mixing ratios (left) and emissions (right) calculated using AFEAS reported end-uses (red) and using a distribution of end-use types as described in the main text. Blue and red lines are medians from posterior distributions, and shaded regions are 1-σ confidence intervals. These posterior estimates do not account for by-product emissions. Note the improved agreement with observations for posterior simulations with uncertain end-uses.

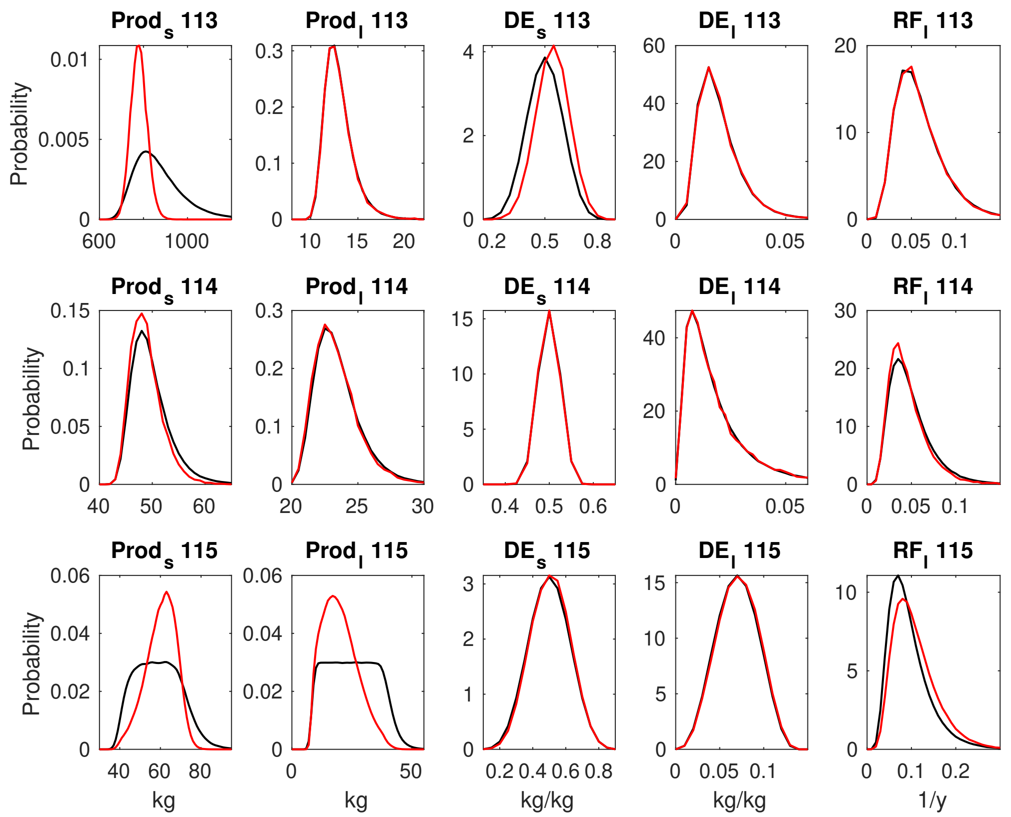

Supplementary Figure 3: Prior (black) and posterior (red) distributions of non-feedstock production for use in short-lived banks (first column) and long-lived banks (second column), the emission rate during the year of production for short-lived banks (third column) and long-lived banks (fourth column), and the annual release rate from long-lived banks (fifth column). All emissions are assumed to occur within two years for short-lived banks, so they are not assigned an annual release rate.

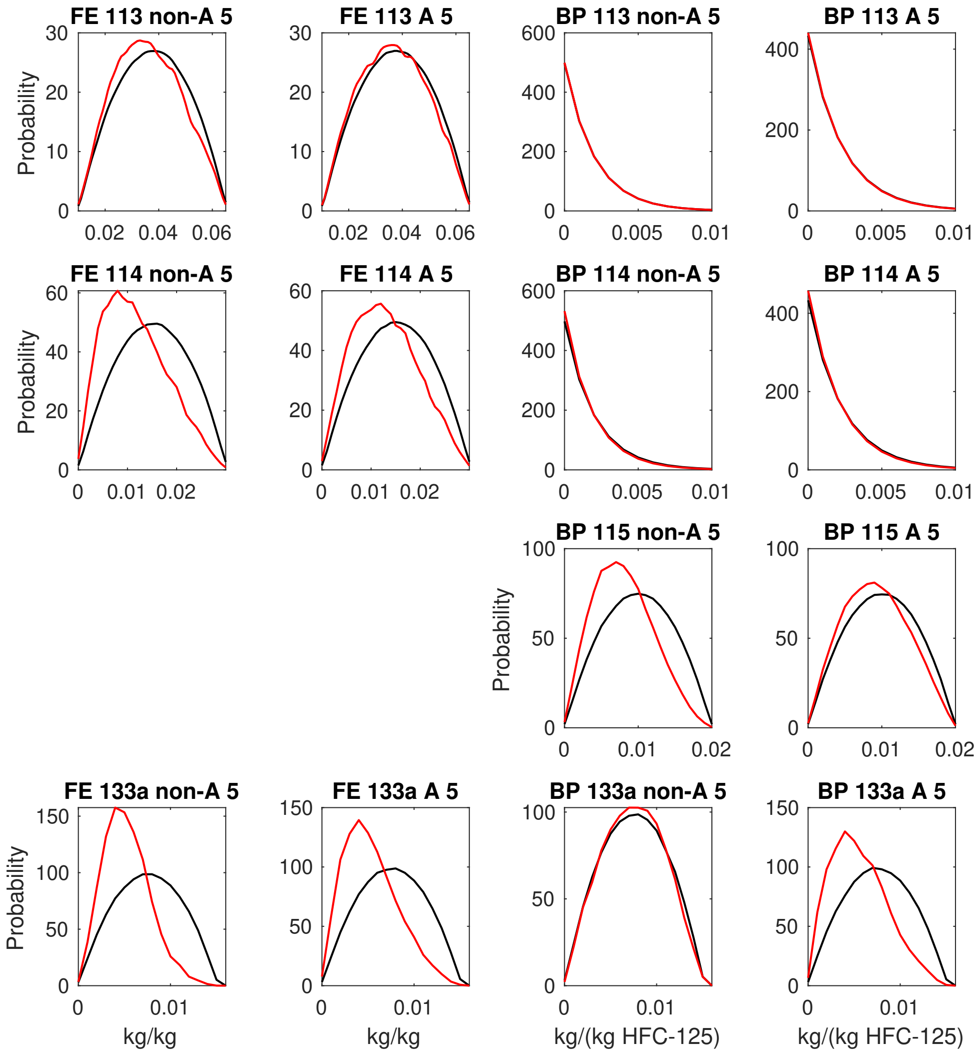

Supplementary Figure 4: Prior (black) and posterior (red) distributions of feedstock emission rates in non-A 5 countries (first column) and A 5 countries (second column) and by-product emission rates in non-A 5 countries (third column) and A 5 countries (fourth column). We assume that CFC-115 is not produced as a feedstock; therefore, it does not have a corresponding feedstock emission rate.

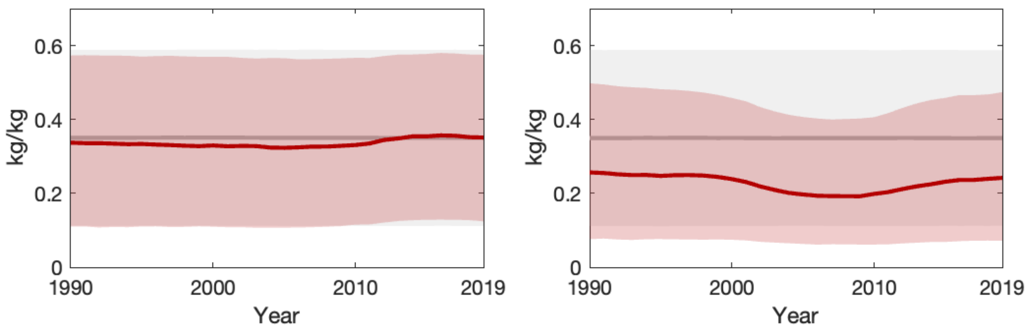

Supplementary Figure 5: Prior (gray) and posterior (red) distributions of the fraction of HFC-134a produced using the PCE pathway (see Fig. 2 in the main text) in non-A 5 countries (left) and A 5 countries (right) from 1990-2019.

Supplementary Tables

Supplementary Table 1: Parameter prior distributions used in mixing ratio simulations. $C_{a \to b}$ refers to the conversion rate from the compound in the given line to the next compound in the chemical manufacturing process.

Species	FE_k	BP_k	$C_{a \to b}$
CFC-113	1.0–6.3%	0.0–0.5*$BP_{115,posterior}$	98%
CFC-114	0.0–3.0%	0.0–0.5*$BP_{115,posterior}$	94%
CFC-115	NA	0.0–2.0%	NA
HCFC-133a	0.0–1.5%	0.0–1.5%	95%

Supplementary Table 2: Median and 1-σ CI of posterior distributions using NOAA CFC-113 surface mixing ratio observations in place of AGAGE CFC-113 data for likelihood calculations. $(1 - \chi)$ is reported for consistency with results presented in the main text (i.e. the fraction of HFC-134a production using the TCE pathway).

Parameter	Global	Non-A 5	A 5
$(1 - \chi)$	74% (59–86%)	80% (57–94%)	67% (43–89%)
FE_{CFC113}	3.4% (2.5–4.4%)	3.4% (2.2–4.7%)	3.5% (2.3–4.8%)
FE_{CFC114}	1.0% (0.5–2.0%)	1.1% (0.5–1.9%)	1.3% (0.6–2.0%)
$FE_{HCFC133a}$	0.5% (0.3–0.7%)	0.5% (0.3–0.8%)	0.5% (0.2–0.8%)
BP_{CFC113}	0.2% (<0.1–0.3%)	0.2% (<0.1–0.4%)	0.2% (<0.1–0.4%)
BP_{CFC114}	0.2% (<0.1–0.3%)	0.1% (<0.1–0.3%)	0.2% (<0.1–0.4%)
BP_{CFC115}	0.8% (0.5–1.0%)	0.8% (0.4–1.3%)	0.9% (0.5–1.4%)
$BP_{HCFC133a}$	0.6% (0.4–0.9%)	0.8% (0.4–1.1%)	0.5% (0.2–0.9%)

Supplementary References

[1] Velders, G. J. M., et al. Projections of hydrofluorocarbon (HFC) emissions and the resulting global warming based on recent trends in observed abundances and current policies. *Atmospheric Chemistry and Physics* **22**, 6087–6101 (2022).

[2] A. J. Sicard, & Baker, R. T. Fluorocarbon refrigerants and their syntheses: Past to present. *Chemical Reviews* **120**, 9164–9303 (2020).

[3] Scott, J. D. & Steven, R. A. Chemical process for the manufacture of 1,1,1,2-tetrafluoroethane. U.S. Patent 5382722A (Jan. 1995).

[4] Vollmer, M. K., et al. Abrupt reversal in emissions and atmospheric abundance of HCFC-133a (CF_3CH_2Cl). *Geophysical Research Letters* **42**, 8702–8710 (2015).

[5] Groppelli, G., Fattore, V., Vecchio, M., & Castellan, A. Catalyst Based on Aluminum Fluoride for the Fluorination in Gaseous Phase of Hydrocarbons. U.S. Patent 3787331 (1974).

[6] Manzer, L. E., & Rao, V. N. M. Process for the Preparation of 1,1,1,2-Tetrafluoroethane. U.S. Patent 50084761 (1991).

[7] McCulloch, A., & Lindley, A. A. From mine to refrigeration: a life cycle inventory analysis of the production of HFC-134a. *International Journal of Refrigeration*,**26**, 865–872 (2003).

[8] Shanthan Rao, P., Narsaiah, B., Rambabu, Y., Sridhar, M., & Raghavan, K. V. Catalytic processes for fluorochemicals: Sustainable alternatives, in *Industrial Catalysis and Separations: Innovations for Process Intensification*, ed. K. V. Raghavan, B. M. Reddy, 407–435, (Apple Academic Press, Toronto, 2015).

[9] Zhang, S., et al. Life cycle assessment and economic analysis of HFC-134a production from natural gas compared with oil-based and coal-based production. *Frontiers of Chemical Science and Engineering* **16**, 1713–1725 (2022).

[10] Vollmer, M. K., et al. Atmospheric histories and emissions of chlorofluorocarbons CFC-13 ($CClF_3$), ΣCFC-114 ($C_2Cl_2F_4$), and CFC-115 (C_2ClF_5). *Atmospheric Chemistry and Physics* **18**, 979–1002 (2018).

[11] Rigby, M., et al. Increase in CFC-11 emissions from eastern China based on atmospheric observations. *Nature* **569**, 546–550 (2019).

[12] Montzka, S. A., et al. A decline in global CFC-11 emissions during 2018-2019. *Nature*, **590**, 428–432 (2021).

[13] Technology and Economic Assessment Panel (TEAP). *Report of the Technology and Economic Assessment Panel Volume 1: Progress Report.* (United Nations Environmental Programme, 2020).

[14] Laube, J. C., et al. Newly detected ozone-depleting substances in the atmosphere. *Nature Geoscience*, **7**, 266–269 (2014).

[15] Vollmer, M. K., et al. Unexpected nascent atmospheric emissions of three ozone-depleting hydrochlorofluorocarbons. *Proceedings of the National Academy of Sciences* **118** (2021), e2010914118.

[16] Nose, M., Takahashi, K., & Shibanuma, T. Method for Producing Pentafluoroethane. U.S. Patent 8975455 (2015).

[17] Medical and Chemical Technical Options Committee (MCTOC). *Report of the Medical and Chemical Technical Options Committee: 2022 Assessment.* (United Nations Environmental Programme, 2022).

[18] Technology and Economic Assessment Panel (TEAP), *Report of the Technology and Economic Assessment Panel Volume 1: Progress Report.* (United Nations Environmental Programme, 2023).

[19] Azzali, D. & Basile, G. Process for Producing 1,1,1,2-Tetrafluoroethane. EP 1153907B1 (2004).

[20] Piepho, E., Wilmet, V., & Buyle, O. Pentafluoroethane Production Method. U.S. Patent 7067707 (June 2006).

[21] Rigby, M., et al. Re-evaluation of the lifetimes of the major CFCs and CH_3CCl_3 using atmospheric trends. *Atmospheric Chemistry and Physics*, **13**, 2691–2702 (2013).

[22] Ko, M. K., et al. Recommended values for steady-state atmospheric lifetimes and their uncertainties. *SPARC Report on the Lifetimes of Stratospheric Ozone-Depleting Substances, Their Replacements, and Related Species*, 6–1 (2013).

[23] Daniel, J. S., et al. Scenarios and Information for Policymakers. In *Scientific Assessment of Ozone Depletion*, **278**, 509 (World Meteorological Organization, 2022).
